# Risk Factors for Prematurity and Congenital Malformations in Assisted Reproductive Technology Pregnancies—A Retrospective Study

**DOI:** 10.3390/jcm13216470

**Published:** 2024-10-29

**Authors:** Raluca Tocariu, Alexandru Dinulescu, Ana Prejmereanu, Călina Maier, Anca-Magdalena Coricovac, Evelyn-Denise Archir, Lucia Elena Niculae, Elvira Brătilă

**Affiliations:** 1 Departments of Obstetrics and Gynecology, Pediatrics, and Anatomy and Embryology, Carol Davila University of Medicine and Pharmacy, 020021 Bucharest, Romania; raluca.tocariu@drd.umfcd.ro (R.T.); calinamaier@yahoo.com (C.M.); anca.coricovac@umfcd.ro (A.-M.C.); elvira.bratila@umfcd.ro (E.B.); 2 Clinical Hospital of Obstetrics and Gynecology Prof. Dr. Panait Sîrbu, 060251 Bucharest, Romania; evelyn-denise.archir@rez.umfcd.ro (E.-D.A.); lucia-elena.ghirca@rez.umfcd.ro (L.E.N.); 3 Emergency Hospital for Children “Grigore Alexandrescu”, 011743 Bucharest, Romania; 4 Gynera Fertility Center, 020308 Bucharest, Romania

**Keywords:** assisted reproductive technology, in vitro fertilization, intracytoplasmic sperm injections, prematurity, congenital malformations

## Abstract

**Background:** Assisted reproductive technology (ART) nowadays plays a major role in the treatment of infertility, with the most frequently used techniques being in vitro fertilization (IVF) and intracytoplasmic sperm injection (ICSI). The objective of this study is to analyze pregnancies achieved using these ART techniques and their correlations with the prematurity and congenital malformations rates. **Methods:** This is an observational retrospective longitudinal study that includes 814 newborns conceived through an ART, namely IVF or ICSI. **Results:** Using a multivariate logistic regression analysis mode, there is a higher prematurity rate in twin pregnancies OR 16 (95% CI 10.7, 23.8), donor conception OR 1.8 (95% CI 1.1, 3.3) and PIH pregnancy OR 2.6 (95% CI 1.5, 4.5). The odds of malformations in these ART pregnancies are increased by the stage of the embryo (day 3) OR 2.6 (95% CI 1.3, 5.2), fresh embryo transfer OR 2 (95% CI 1.2, 3.4) and donor conception OR 2.3 (95% CI 1.2, 4.4). The ART used (IVF/ICSI) does not influence the prematurity or birth defects rate. **Conclusions:** Donor conception is found to increase the odds of both prematurity and congenital malformations. The ART used (IVF/ICSI) does not influence the prematurity or birth defects rate.

## 1. Introduction

Assisted reproductive technology (ART) nowadays plays a major role in the treatment of infertility, alongside pharmacological therapy and surgical therapy. Since its first success in 1978, ART has accounted for millions on births worldwide, showing great progress in the last decade and expanding its indications beyond its original purpose [1,2]. The concept of ART refers to medical procedures that involve handling eggs or embryos outside a female’s body to achieve a pregnancy, and it was firstly developed to treat infertility due to various causes, like tubal factor infertility, male factor infertility, diminished ovarian reserve, ovarian failure or dysfunction [3,4]. The most common definition of infertility considers a 12-month periods of not achieving a pregnancy despite unprotected sexual intercourse [5]. Besides infertility, other indications for ART include preimplantation genetic testing, fertility preservation and patients desiring to delay childbearing [4].

**Figure 1:**
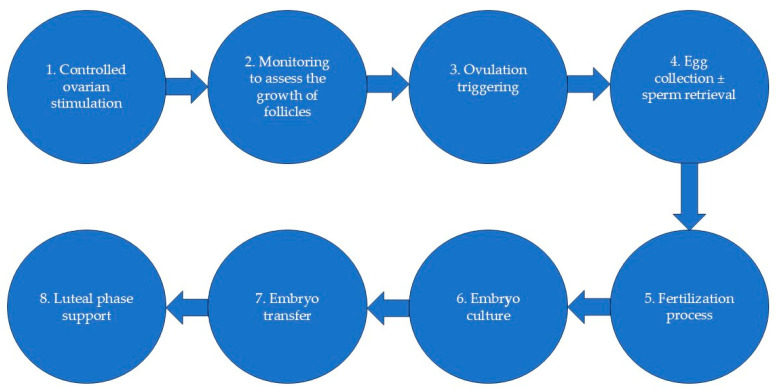
ART cycle.

ART is a complex process that involves a series of sequences known as an ART cycle. The first step consists of controlled ovarian stimulation, with three main objectives: hypophyseal activity suppression, multiple follicle growth stimulation and ovulation induction, with the production of 5 to 10 mature eggs, but the number of eggs collected will in reality vary depending on the woman’s ovarian reserve and response to stimulation [6,7]. The cycle continues with the egg retrieval stage, in which the eggs are surgically removed from the ovaries; then, the eggs are combined with the sperm in a laboratory (Figure 1) [6,7,8]. If the fertilization process succeeds, the embryo transfer stage follows, using a fresh or frozen embryo, with recent studies showing better pregnancy outcomes using frozen embryo transfer [9,10]. The contemporary possibilities of ART include in vitro fertilization (IVF), embryo transfer (ET), gamete or zygote intrafallopian transfer (GIFT or ZIFT), intracytoplasmic sperm injection (ICSI), embryo biopsy, preimplantation genetic testing, assisted hatching and cryopreservation of embryos or gametes. Among these techniques, the most frequently used are IVF and ICSI [11,12]. Both IVF and ICSI are laboratory procedures that involve aspiration of oocytes from the follicles. The difference between them lies in how insemination occurs. In IVF, the sperm is placed on a Petri dish with the woman’s eggs and the selection of the spermatozoa that fertilize the oocyte happens naturally, while in ICSI, a single sperm is selected from a semen sample and injected into the oocyte [13,14]. ICSI has become the method of choice in couples with severe male-factor infertility, but it has no obvious advantage over conventional IVF in patients with normal semen parameters [15,16].

The complexity of the process involves a series of complications, from short-term to long-term ones, affecting both the mother and the child. The maternal complications include thromboembolic events, mainly in the upper limbs, head and neck, obstetric complications such as a higher risk of caesarian section, gestational diabetes and placental abnormalities and malignancies. The fetal and neonatal complications are represented by imprinting disorders, a higher risk of preterm birth, low birth weight and congenital malformations; for example, cardiac, gastrointestinal and genitourinary defects. The majority of the adverse outcomes resulting from ART are caused by multiple gestation [3,17,18,19,20].

The objective of this study is to analyze pregnancies achieved using ART, including their associated complications, and to analyze the risk factors in these pregnancies for prematurity and for congenital malformations.

## 2. Materials and Methods

This is an observational retrospective longitudinal study that includes 814 newborns who came from female patients who underwent an ART, namely IVF or ICSI, at the Clinical Hospital of Obstetrics Prof. Dr. Panait Sîrbu in Bucharest, Romania and at the Gynera Fertility Center in Bucharest, Romania, between January 2020 and December 2023.

### 2.1. Eligibility Criteria

The inclusion criteria were as follows: women who came from couples that suffered from infertility and underwent an ART (IVF/ICSI). The transferred embryos were fresh or frozen, the stage of the embryo was day 3 or day 5, and the oocytes were their own or donated. Prematurity was defined as a gestational age lower than 37 weeks.

### 2.2. Data Collection

The data were collected in Microsoft Excel 2016 from the electronic register of the hospital and the patient charts. The data collected were the age of the patients, infertility etiology, pregnancy complications, the ART used, the maturity of the embryos and the newborn’s data (gestational age and the presence of malformations). There was no control group used. The bias was reduced through simple random sampling.

### 2.3. Statistical Analyses

The sample size was calculated using the following formula:

$$n=\frac{z^{2}*\hat{p}(1-\hat{p})}{\varepsilon^{2}}$$

where n is the sample size; z is the z-score; $\hat{p}$ is the population proportion; and ε is the margin of error (confidence interval). 

The sample size was calculated with a confidence interval of 95% and a margin error of ±0.68%

We considered a *p* value ≤ 0.005 for data significance.

The analysis was performed using IBM SPSS version 26. The Shapiro–Wilk test was used to analyze the quantitative variables’ distribution, and they were reported as averages with standard deviations or medians with interquartile ranges according to their distribution. Fisher’s exact test was used to determine if there were nonrandom associations between two categorical variables. The logistic regression models were verified for the goodness of fit and used for estimating the prediction value of twin pregnancy, PIH pregnancy, IVF pregnancy and donor conception (in the univariate and multivariate models) for prematurity and the prediction value of the day of the embryo, protocol for embryo transfer and donor conception (in univariate and multivariate models) for congenital malformations.

## 3. Results

### 3.1. Prematurity

A total of 267 (32.8%) of the newborns were preterm.

The age of the women who had undergone ART did not follow a normal distribution (*p* < 0.001) and had a median of 35 (32–38) years. The prematurity rate was not influenced by the age of the mothers (*p* = 0.624).

The etiology of the infertility rate was similar from female (36.2%) and both partners causes (34.6%) and lower from male causes (29.1%). The prematurity rate was not influenced by the infertility etiology by sex (*p* = 0.805).

A total of 220 (27%) of the newborns were from twin pregnancies, there were no multiple pregnancies with more than two fetuses, and the twin pregnancies rate in this study was equivalent to the multiple pregnancies rate. The rate of prematurity was much higher in the twin pregnancies (Table 1).

Moreover, 110 (13.5%) of the newborns came from gestational diabetes mellitus (GDM) pregnancies and 95 (11.7%) came from pregnancy-induced hypertension (PIH) pregnancies. There was no influence of the prematurity rate by GDM (***p*** = 0.913), but the rate was higher in the PIH pregnancies (Table 2).

In terms of the ART used, the majority of the newborns came from IVF pregnancies (65.8%). The rate of prematurity was higher in the pregnancies that came from IVF (Table 3).

The majority of the embryo transfers, 594 (73%), were performed in an artificial cycle and 220 (27%) in a natural cycle. The prematurity rate was not influenced by the embryo transfer type of cycle (*p* = 0.402).

Furthermore, 75 (9.22%) of them came from donor conception. The prematurity rate was higher in the pregnancies that came from donor conception (Table 4).

Most of the embryo transfers were performed with frozen embryos (77.6%) (632 frozen and 182 fresh) and with day 5 embryos (92.6%). The prematurity rate was not influenced by the protocol for embryo transfer, frozen/fresh (*p* = 0.720) or the stage of the embryo, D3/D5 (*p* = 0.391).

We found an association between the prematurity rate and twin pregnancies, PIH, IVF and donor conception.

The data from Table 5 represent the logistic regression models for prematurity. In the univariate models, all the included variables had a significant predictive effect on the weight of children:

- Having a twin pregnancy increased the odds of prematurity by 1456% (95% CI: 968.3–2268%).
- Having a PIH pregnancy increased the odds of prematurity by 122.1% (95% CI: 44.1–242.2%).
- Having an IFV pregnancy (vs. ICSI pregnancy) increased the odds of prematurity by 72.7% (95% CI: 25–138.5%).
- Having a donor conception increased the odds of prematurity by 249.3% (95% CI: 114.4–469.1%).

A multivariate regression model was constructed using the variables included in the univariate models. In this model, only IFV pregnancy did not have a significant effect:

- Having a twin pregnancy increased the odds of prematurity by 1501% (95% CI: 974–2289%).
- Having a PIH pregnancy increased the odds of prematurity by 167.6% (95% CI: 58.4–352%).
- Having a donor conception increased the odds of prematurity by 80.4% (95% CI: 18.6–230.2%).

### 3.2. Congenital Malformations

A total of 78 (9.6%) had congenital malformations.

There was no correlation between congenital malformations and the age of the mother (*p* = 0.376), GDM (*p* = 0.161), PIH (*p* = 0.063), twin pregnancies (0.348), ART technique (0.616), infertility etiology (*p* = 0.567) and type of cycle (*p* = 0.893).

The rate of congenital malformations was higher in the day 3 embryos (Table 6).

The rate of congenital malformations was higher in the fresh embryos (Table 7).

The rate of congenital malformations was higher in the pregnancies that came from donor conception (Table 8).

We found an association between the rate of congenital malformations and the stage of the embryo, the protocol for embryo transfer and donor conception.

The data from Table 9 represent the logistic regression models for the congenital malformations rate. In the univariate models, all the included variables had a significant predictive effect on the weight of the children:

- Having a day 3 embryo increased the odds of congenital malformations by 228.1% (95% CI: 71.2–529%).
- Having a fresh embryo increased the odds of congenital malformations by 122.5% (95% CI: 37.8–269%).
- Having a donor conception increased the odds of congenital malformations by 142.1% (95% CI: 28.3–356.7%).

A multivariate regression model was constructed using the variables included in the univariate models. All the variables had a significant effect.

- Having a day 3 embryo increased the odds of congenital malformations by 166.4% (95% CI: 36.2–420.8%).
- Having a fresh embryo increased the odds of congenital malformations by 108.7% (95% CI: 25.9–246%).
- Having a donor conception increased the odds of congenital malformations by 132.3% (95% CI: 21.2–345.4%).

## 4. Discussion

Prematurity or preterm is defined by the World Health Organization (WHO) as a birth before 37 completed weeks of pregnancy. The global prevalence of prematurity was estimated to be 9.9% (95% CI: 9.1–11.2%) in 2020 [21,22]. The preterm rate reported in this study was significantly higher than the global prevalence, 32.8%. The most important factor for prematurity in the multivariate logistic regression analysis model was twin pregnancy, increasing the odds of prematurity by almost 16 times (*p* < 0.001). Excluding the twin pregnancies, the preterm rate in the singleton pregnancies in this study was 16.8%, almost three times higher than the prematurity rate found in spontaneously conceived singletons (4.74–5.5%), meaning that ART may be considered an independent factor for preterm births. The preterm rate in this study was also higher than the singleton ART pregnancy rate found in the literature (7.13–12.8%) [23,24,25]. Other independent factors found in this model were donor conception, increasing the odds by ~1.8 times (*p* = 0.044), and PIH pregnancy, with an increase of 2.6 times (*p* < 0.001). PIH is a known factor for prematurity and other neonatal complications, and several studies found in the literature describe a higher risk of PIH in ART pregnancy, meaning that ART pregnancy can also be considered an indirect risk factor for prematurity through the increased risk of PIH [26,27,28,29,30,31,32].

Congenital abnormalities, also known as congenital malformations or birth defects, are structural or functional anomalies defined as occurring during intrauterine life. A me-ta-analysis published by Veeramani et al. described ART pregnancy as having a higher risk of malformations (OR 0.67; *p* < 0.001) than the naturally conceived ones and one published by Klonoff-Cohen and Polavarapu described a higher rate in IVF than in naturally conceived ones. In this study, the prevalence of congenital malformations was 3.2–4.8 higher than the global prevalence (9.6% vs. 2–3%) [33,34,35,36]. In the multivariate logistic regression analysis model constructed in this study, it was found that using a day 3 embryo (vs. a day 5 embryo) increased the odds of congenital malformations by 2.6 times (*p* = 0.004), the fresh type (vs. frozen) increased the odds by 2 times (*p* = 0.004) and donor conception pregnancy increased the odds by 2.3 times (*p* = 0.011).

Infertility is a global public health problem caused by both female and male factors, but in some cases the cause remains unknown [37]. In this study, the prevalence of female etiology and both partners was similar, 36.2% and 34.6%, respectively, and lower from male causes (29.1%). The prevalence of both partners and the male causes was in accordance with the prevalence reported by the European Society of Human Reproduction and Embryology and the female etiology prevalence was slightly higher (36.2% vs. 20–35%) [38]. The prematurity rate or the rate of congenital abnormalities was not influenced by the infertility etiology (*p* = 0.805; *p* = 0.567).

Nowadays, ICSI has become the most widely used insemination method worldwide. In Europe, in 2014, the majority (71.3%) of fresh IVF/ICSI cycles were performed with ICSI, but it is still of debate whether ICSI is a better option than standard IVF. A randomized controlled trial conducted by Bhattacharya et al. showed that ICSI offers no advantage over conventional IVF in terms of the clinical outcome in couples with non-male-factor infertility [39,40,41,42]. The findings on whether the type of ART influences the risk of preterm birth are inconsistent. A meta-analysis conducted by Pinborg et al. showed a lower risk of preterm birth in singletons when using ICSI compared to traditional IVF [43]; however, other studies showed that the risk of preterm birth is not affected by the type of ART procedure, which is in accordance with our study, which in the multivariate logistic regression analysis model found that the type of ART (IVF/ICSI) did not increase the odds of prematurity (*p* = 0.528). Another inconsistent finding from the data in the literature is the correlation between the type of ART and the rate of congenital malformations. A very large cohort study (32.484 fresh ICSI vs. 47.178 fresh IVF) by Henningsen et al., which used the Committee of Nordic Assisted Reproductive Technology and Safety (CoNARTaS) database, found a higher risk of malformations in fresh ICSI than in fresh IVF (6% vs. 5.3). A meta-analysis by Veeramani et al. described a 9% higher chance in those conceived through IVF vs. ICSI (*p* = 0.006). However, two retrospective cohort studies and a meta-analysis performed by Wen et al. described no difference, the same as our study, which found that the ART technique did not influence the malformations rate (*p* = 0.616) [33,44,45,46,47].

Singleton pregnancies resulting from donor oocytes are more likely to be preterm than those resulting from autologous oocytes [48,49,50,51]. This result was also found in our study, as mentioned above, in which the donor conception pregnancies had doubly increased odds of prematurity. Searching the literature, we found two studies that claimed there is no relation between donor oocytes and an increased risk of congenital malformations, but in our study, the birth defects rate was increased by 1.8 times in the pregnancies that resulted from donor oocytes [51,52].

Many meta-analyses found in the literature describe how frozen embryo transfer has a lower risk of prematurity than fresh embryo transfer [53,54,55]. In this study, the preterm rate was not influenced by the protocol for embryo transfer or by the use of fresh or frozen embryos (*p* = 0.720). About the malformations risk, the studies found in the literature are inconsistent, as several studies described no difference in the birth defects rate by the protocol for embryo transfer and one study performed by Hwang et al. reported an increased risk of congenital anomalies in those born from frozen embryo transfer [46,55,56,57,58]. Our study reports the opposite to both variants found in the literature, namely a double risk of congenital malformations in those conceived through fresh embryo transfer (*p* = 0.004).

The stage of the embryo (day 3/day 5) is known to influence the prematurity rate, with a pregnancy that uses blastocysts (day 5) having an increased risk of preterm newborns [25,59,60]. The stage of the embryo did not influence the prematurity rate in this study (*p* = 0.391). We found no study that described the influence of the embryo stage on the malformations rate. In this study, as was aforementioned, the day 3 embryo pregnancies had 2.6 times (*p* = 0.004) increased odds of congenital malformations in the multivariate logistic regression analysis model.

This study has several limitations that should be mentioned. We lacked data about the malformation type, number of embryos transferred, Gardner classification, type of stimulation drug, total dose of FSH and protocol type (agonist vs. antagonist), and the study was performed in the time of the COVID-19 pandemic. With placental SARS-CoV-2 infection having been proven, both the mother and the fetus can be affected by the virus. A higher rate of preeclampsia, preterm delivery and C-section was shown in IVF or ICSI pregnancies with SARS-CoV-2 infection compared with ART pregnancies that were not affected by the virus. A small percentage of neonates had respiratory symptoms at birth [61,62,63,64,65].

## 5. Conclusions

The prematurity rate reported in this study was three times higher than the global prevalence. The ART used (IVF/ICSI) did not influence the prematurity or birth defects rate. The independent factors found to increase the prematurity rate in ART pregnancies were twin pregnancy, PIH, and donor conception, and the ones that increased the malformations rate were the use of day 3 embryos, fresh embryos and donor conception. Donor conception was found to increase the odds of both prematurity and congenital malformations.

## Figures and Tables

**Table 1:** Rate of prematurity in single pregnancies vs. twin pregnancies.

	Term Newborns	Preterm Newborns	Fisher’s Exact Test (*p*)
Single pregnancy	494 (83.2%)	100 (16.8%)	<0.001
Twin pregnancy	53 (24.1%)	167 (75.9%)	

**Table 2:** Rate of prematurity in non-hypertension pregnancies vs. PIH pregnancies.

	Term Newborns	Preterm Newborns	Fisher’s Exact Test (*p*)
Non-hypertension pregnancy	499 (69.4%)	220 (30.6%)	<0.001
PIH pregnancy	48 (50.5%)	47 (49.5%)	

**Table 3:** Rate of prematurity by ART.

	Term Newborns	Preterm Newborns	Fisher’s Exact Test (*p*)
IVF pregnancy	339 (63.2%)	197 (36.8%)	=0.001
ICSI pregnancy	208 (74.8%)	70 (25.2%)	

**Table 4:** Prematurity rate by donor conception.

	Term Newborns	Preterm Newborns	Fisher’s Exact Test (*p*)
No	517 (70%)	222 (30%)	<0.001
Yes	30 (40%)	45 (60%)	

**Table 5:** Logistic regression for prematurity.

Parameter	Univariable		Multivariable	
	OR (95% CI)	*p*	OR (95% CI)	*p*
Twin pregnancy	15.556 (10.683–22.680)	**<0.001**	16.018 (10.74–23.889)	**<0.001**
PIH pregnancy	2.221 (1.441–3.422)	**<0.001**	2.676 (1.584–4.52)	**<0.001**
IVF pregnancy	1.727 (1.25–2.385)	**=0.001**	0.881 (0.595–1.306)	**=0.528**
Donor conception	3.493 (2.144–5.691)	**<0.001**	1.804 (1.186–3.302)	**=0.044**

**Table 6:** Rate of congenital malformations by the stage of the embryo.

	Day 3 Embryo	Day 5 Embryo	Fisher’s Exact Test (*p*)
Normal newborn	46 (76.7%)	690 (91.5%)	=0.001
Congenital malformations	14 (23.3%)	64 (8.5%)	

**Table 7:** Rate of congenital malformations by the protocol for embryo transfer (fresh/frozen) of the embryo.

	Fresh Embryo	Frozen Embryo	Fisher’s Exact Test (*p*)
Normal newborn	153 (84.1%)	583 (92.2%)	=0.002
Congenital malformations	29 (15.9%)	49(7.8%)	

**Table 8:** Rate of congenital malformations by donor conception.

	Own Conception	Donor Conception	Fisher’s Exact Test (*p*)
Normal newborn	675 (91.3%)	61 (81.3%)	=0.011
Congenital malformations	64 (8.7%)	14 (18.7%)	

**Table 9:** Logistic regression for congenital malformations.

Parameter	Univariable		Multivariable	
	OR (95% CI)	*p*	OR (95% CI)	*p*
Stage of embryo (day 3)	3.281 (1.712–6.29)	**<0.001**	2.664 (1.362–5.208)	**0.004**
Protocol for embryo transfer (fresh)	2.255 (1.378–3.69)	**<0.001**	2.087 (1.259–3.46)	**0.004**
Donor conception	2.421 (1.283–4.567)	**=0.006**	2.323 (1.212–4.454)	**0.011**

## Data Availability

The data can be shared up on request.
